# The impact of school heavy metal exposure on children's gut microbiota: The mediating role of environmental microorganisms

**DOI:** 10.1002/imt2.70021

**Published:** 2025-03-25

**Authors:** Yuchen Zou, Menglong Li, Tuerxunayi Abudumijiti, Huiming He, Mengying Guan, Yeerlin Asihaer, Miao Li, Nourhan M. Khattab, Mushui Shu, Yifei Hu

**Affiliations:** ^1^ Department of Child, Adolescent Health and Maternal Care, School of Public Health Capital Medical University Beijing China; ^2^ Institute of Urban Safety and Environmental Science, Beijing Academy of Science and Technology Beijing China

## Abstract

Heavy metals are toxic and harmful pollutants that can affect the school environment and the exposed children's health. This study collected dust samples and children's fecal specimens, and performed gene sequencing. We used eXtreme Gradient Boosting to determine the impact of heavy metals on environmental microorganisms and gut microbiota, while using the relative length of the quadrant and Fourth‐corner analysis to explore the relationship among the three components. We found heavy metal pollution existed in the classroom environment, with lead and copper significantly affecting environmental microorganisms' community structure. Although nonsignificant associations were observed between heavy metals and gut microbiota, Fourth‐corner analysis revealed the associations were significantly mediated by environmental microorganisms. Both heavy metals and microorganisms in the environment can disrupt the microbial community structure in the intestines of exposed children.

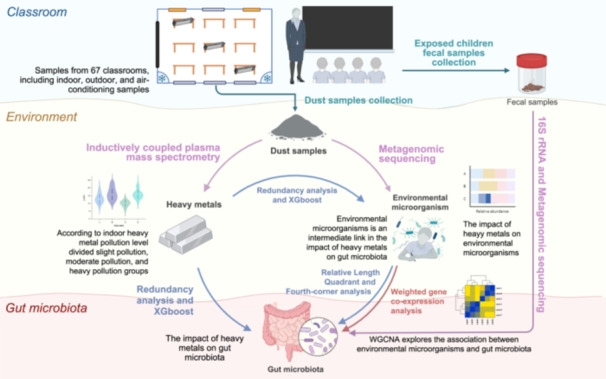

Heavy metals are toxic environmental pollutants that have garnered public attention [1]. The sensitivity of school‐age children to their external environment increases their susceptibility to heavy metal hazards [2, 3, 4]. Heavy metal pollution significantly affects the diversity of microorganisms in the environment [5, 6]. Environmental microorganisms, which can be impacted by heavy metals, may enter the human body through various pathways, ultimately reaching the gut. There, they can modify the microbiota composition, disrupt its balance, and impair host health [7]. However, research on the interactions among heavy metals, environmental microorganisms, and gut microbiota remains limited. We analyzed heavy metal [cadmium (Cd), cobalt (Co), chromium (Cr), copper (Cu), manganese (Mn), nickel (Ni), lead (Pb), and vanadium (V)] content in classroom dust samples to evaluate the classroom environmental quality and risk characteristics of primary schools, and sequenced environmental microorganisms and gut microbiota. This study investigates the impact of heavy metals in school environments on environmental microorganisms, the gut microbiota of exposed children, and the interactions among these three factors. The findings can offer insights for targeted interventions to facilitate healthy school development.

## RESULTS AND DISCUSSION

### Heavy metal pollution existed in classroom environment

**Figure 1:**
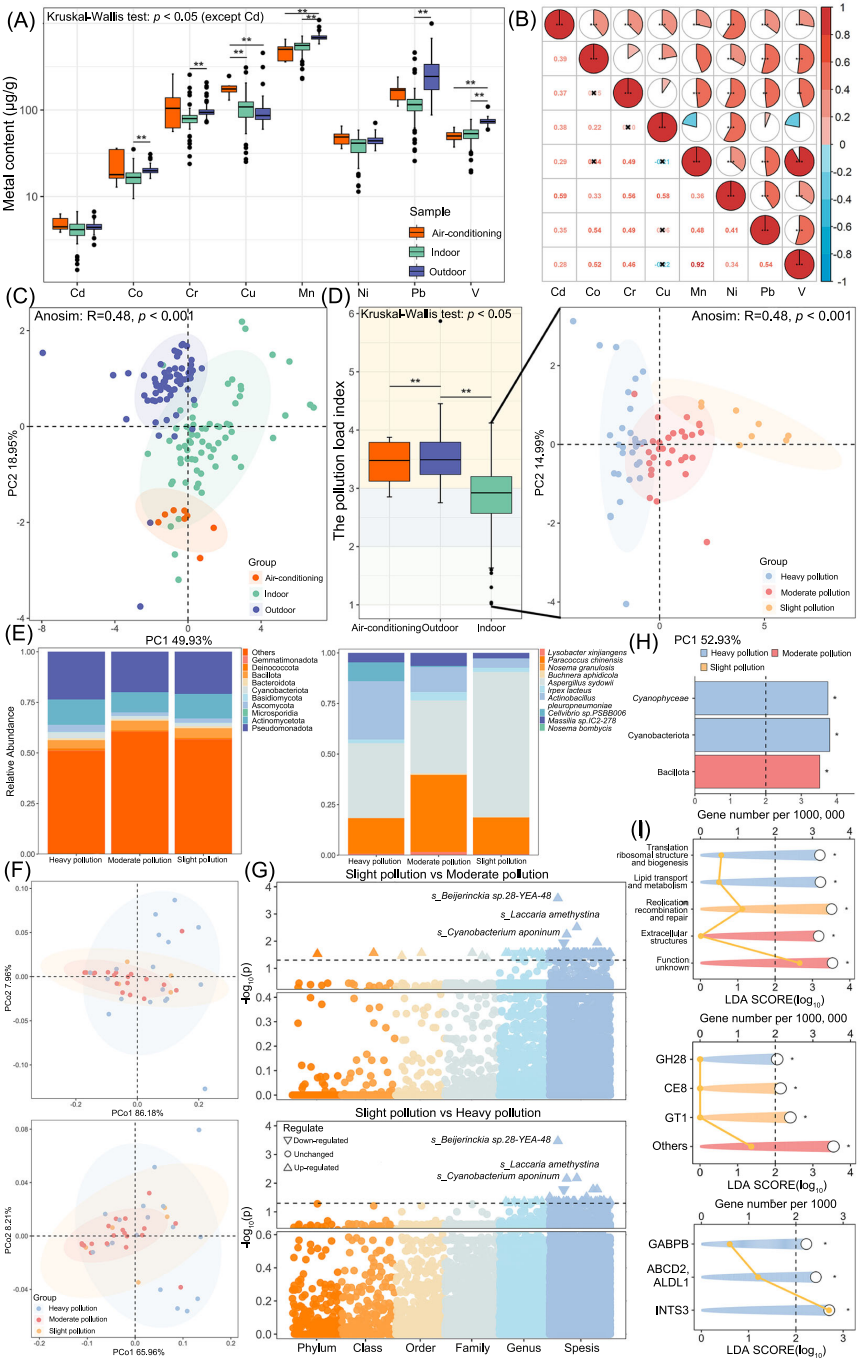
Heavy metal and indoor microorganisms' distribution. (A) The concentration of heavy metals at different sampling locations. (B) Spearman autocorrelation of heavy metal: the lower triangle of the correlation matrix displays the correlation coefficient, with a cross indicating no significating non‐significant correlations; the upper triangle presents the fan‐shaped correlation coefficient. (C) Principal component analysis (PCA) of heavy metals intercorrelations, with a 95% confidence interval (CI) for shadows. (D) Pollution load index of three sampling locations (left) and PCA of indoor samples (right). The *p* of the Kruskal–Wallis test was corrected by the false discovery rate (FDR). (E) The indoor microorganism's composition in different pollution levels at the phylum‐level (left), and the species‐level (right). (F) Principal co‐ordinates analysis (PCoA) at the phylum‐level (upper) and species‐level (lower) of indoor microorganisms. (G) Differential indoor microorganisms identified by the fold change (FC) method, different colors represent distinct taxonomy; up‐triangles indicate an increased abundance compared to slight pollution, while down‐triangles indicate a decreased abundance. (H) The Linear discriminant analysis Effect Size (LEfSe) analysis results for microorganisms. (I) The LEfSe analysis results for homologous gene clusters (upper), carbohydrate enzymes (middle), and Kyoto Encyclopedia of Genes and Genomes, homologous gene clusters (lower). The yellow line represents the number of annotated genes displayed on the upper *X*‐axis. Statistical significance was assessed using 999 permutation tests. *p < 0.05, **p < 0.01, ***p < 0.001. Air‐conditioning was the air‐conditioner filter sample.

Mn exhibited the highest concentration of metal elements, while Cd had the lowest concentration in dust (Table S1, Figure 1A), and strong positive intercorrelations were observed among all heavy metals (Figure 1B). Significant differences in the composition of heavy metals were determined in dust from different sites (*p* < 0.001) (Table S2, Figure 1C). For the pollution evaluation, the pollution load index (PLI) of indoor dust was significantly lower than that of air‐conditioner filter and outdoor (*p* < 0.05) (Figure 1D). In addition, the average Geoaccumulation index and Enrichment factor indicated a heavy pollution of Cd (4.98 and 52.24, respectively) (Figure S1).

### Environmental microorganisms and gut microbiota exhibit changes across varying pollution levels

The species accumulation and rank abundance curves showed sufficient sequencing depth and comprehensive sampling (Figure S2A,B). The absolute abundance of microorganisms is shown in Figures S3 and S4. The proportion of major microorganisms in phylum‐level and species‐level varies obviously among the three sites (Figure S5A). Although Alpha analysis showed no significant difference (Figure S2C), principal co‐ordinates analysis (PCoA) results presented significant separation of microbial structures across different sites (*p* < 0.001) (Figure S5B). The Linear discriminant analysis Effect Size (LEfSe) differential analysis results are shown in Table S3 and Figure S5C. In the Kyoto Encyclopedia of Genes and Genomes (KEGG) functions analysis, methyl‐accepting chemotaxis protein (MCP) was enriched [linear discriminant analysis (LDA) value = 3.08, *p* < 0.001] (Figure S5D). MCP was enriched as a source element in the Bacterial Chemotaxis KEGG map (Figure S6), indicating heavy metals affected the response of environmental microorganisms to external stimuli, consistent with the findings of previous studies [8, 9, 10]. Other function analysis results were showed in Figure S5E,F. Furthermore, at both phylum‐ and species‐levels, clear differences in relative abundance of indoor microorganisms were observed across varying pollution levels. *Actinobacillus_pleuropneumoniae* showed a higher abundance in indoors heavy pollution (Figure 1E). The primary virulence factors of *Actinobacillus_pleuropneumoniae* are RTX (Apx) toxins, which exhibit strong hemolytic and cytotoxic effects. These toxins can induce damage and apoptosis of alveolar macrophages, compromising pulmonary immune defense and leading to respiratory complications in exposed individuals [11, 12]. Alpha analysis (Figure S2D) and PCoA results (Figure 1F) showed no significant differences across three pollution levels. Compared to slight pollution, there were 155 microorganisms presented significantly higher abundance in moderate pollution and 48 microorganisms in heavy pollution [|log_2_ fold change (FC) < 2|, log_10_ *p* < 0.05] (Table S4, Figure 1G). LEfSe analysis further highlighted that Cyanobacteriota was a key taxon in heavy pollution (LDA = 3.80, *p* < 0.05) (Figure 1H). Detailed microbial functional differences are shown in Figure 1I.

The quality of gut microbiota 16 s rRNA sequencing was presented in Figure S7A,B. All heavy metals posed no health risks yet (Figure S8A). The relative abundance at phylum‐ and genus‐levels is shown in Figure S8B. Alpha analysis (Figure S7C,D) and PCoA results (Figure S8C) showed no significance across three pollution levels. As for the differential analysis, FC methods results were shown in Figure S8D; the LEfSe analysis indicated that *Escherichia*_*Shigella* showed higher abundant in moderate pollution (LDA = 3.92, *p* < 0.05); *Leuconostocaceae*/*Weissella* showed higher abundant in heavy pollution (LDA = 3.46, *p* < 0.05) (Figure S8E). Differential gut microbiota is mainly enriched in Galactose PTS system EIIC component, Uncharacterized N‐acetyltransferase, and Toxin CcdB (Figure S8F). Differential KEGG Orthology in KEGG map is shown in Table S5 and Figure S6. By examining the metabolic pathways, interactions, and environmental response mechanisms of microorganisms from a comprehensive perspective, we can clarify their critical roles in a wide range of biological processes and identify potential adverse effects on children's health.

### Specific heavy metals affect environmental and gut microbial community structure and diversity

**Figure 2:**
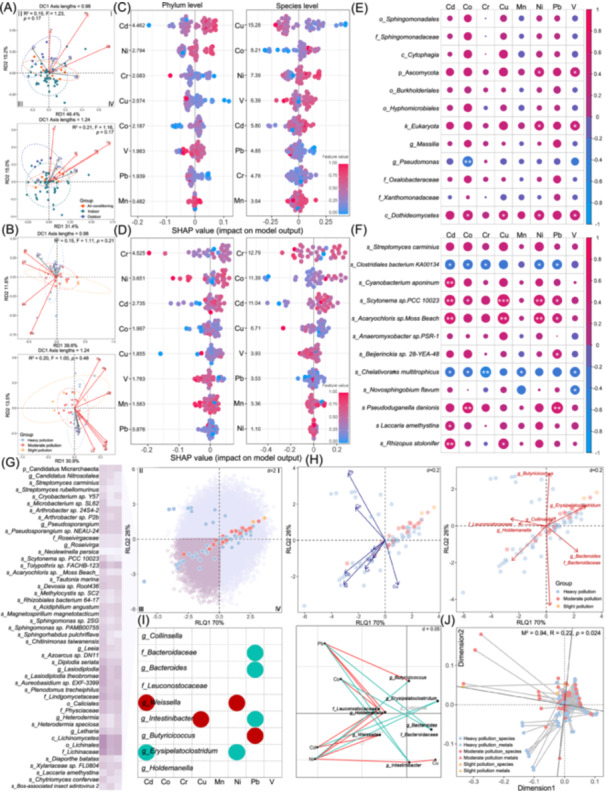
The impact of heavy metals on environmental microorganisms and exposed children gut microbiota. Redundancy Analysis (RDA) results at the phylum‐ level (upper) and species‐level (lower) of environmental microorganisms (A) and indoor microorganisms (B). The title is the Detrended Correspondence Analysis result of the longest axis of the trend; the ellipse represents the 95% confidence intervals; variables with high correlation show consistent arrow directions, and arrow lengths represent their ranking contributions. Red arrows denote significant heavy metals. SHapley Additive exPlanations (SHAP) analysis of heavy metals and environmental microorganisms (C) or indoor microorganisms (D) Shannon index. The number on the right side of the *Y*‐axis represents the importance of index; the red indicates high heavy metal concentration, while the blue indicates low. Spearman's rank correlation between heavy metals and site differential microorganisms (E) or pollution differential microorganisms (F) and heavy metals. (G) Sample and environmental microbial matrix (right) and 47 differential gut microbiotas located in the third quadrant (left). (H) The spatial contribution of heavy metals (left) and differential gut microbiota (right), where variables with high correlation tend to have consistent arrow directions, and the length of the arrows represents the degree of contribution of the variables to the ranking. (I) Fourth‐corner analysis results of the FDR method for adjusted‐*p* (left) and original‐*p* (right), a significant positive correlation is red, and a significant negative correlation is cyan. (J) Procrustes Analysis results of heavy metals and differential gut microbiota. Each segment represents a sample, with a solid circle representing gut microbiota data, and a triangle at the other end representing the heavy metal data; the line represents the vector residual of two sorted configurations, and the shorter the line, the higher the consistency between the datasets; statistical significance was evaluated using 999 permutation tests. *p < 0.05, **p < 0.01, ***p < 0.001. Air‐conditioning was the air‐conditioner filter sample. FDR, false discovery rate.

Pb and Co significantly affected environmental and indoor microbial structure (all *p* < 0.05) (Table S6, Figure 2A,B). The SHapley Additive exPlanations (SHAP) results showed that Cd affected the Shannon index at the phylum‐level, Cu affected the species‐level across all sites (Figure 2C), and Cd affected the indoor sample's Shannon index (Figure 2D). All metals negatively impacted microbial diversity at the phylum‐ and species‐levels, except for Ni and Cu at the species‐level (Figure S9). Correlation analysis indicated that the correlation between key heavy metals and pollution‐associated microorganisms was stronger than that with site‐associated microorganisms (Figure 2E,F). Functional analysis further showed that indoor microorganisms' functions were affected by Pb and Co (all *p* < 0.05) (Figure S10A). As the pollution level increased, the samples shifted toward the fourth quadrant, reflecting the influence of heavy metals (Table S6, Figure S10B). Mantel analysis of all sites' samples suggested that Co significantly affected all KEGG level1 functions (all *p* < 0.05) (Table S7, Figure S10C), though the effect was not significant in indoor samples (Figure S10D). Pb and Cd significantly impact school environmental microorganisms; studies have shown that Pb or Cd pollution could exacerbate structure and function changes [13, 14, 15, 16].

For gut microbiota, Pb, Cu, Cd, Co, and V presented significant impact at the genus‐level (all *p* < 0.05) (Table S6, Figure S11A). The SHAP analysis indicated that Cr, Co, and Cd affected the Shannon index at the phylum‐level; Mn, V, and Cr affected at the genus‐level (Figure S11B). Only *Collinsella* showed significant positive correlation with Mn (*r*_*s*_ = 0.15), Ni (*r*_*s*_ = 0.11), and V (*r*_*s*_ = 0.13) (all *p* < 0.05) (Figure S11C); only Cu significantly affected microbial KEGG function (*p* < 0.05) (Table S6, Figure S11D). Mantel analysis found that Co significantly affected the environmental information processing pathway (*r* = 0.25, *p* < 0.05), Cr affected organic systems (*r* = 0.71, *p* < 0.01), and Pb affected cellular processes (*r* = 0.49, *p* < 0.05) (Table S7, Figure S11E). Overall, the correlations between specific heavy metals and gut microbiota or microbial functions were more subtle than that of environmental microorganisms.

### Environmental microorganism is the mediator between heavy metals and gut microbiota

In the relative length of the quadrant analysis for samples and species abundance matrix, as the pollution level increased, the dots moved towards the third quadrant, where 47 upregulated environmental microorganisms were located (Figure 2G). Ni, Cd, Cr, Mn, V, and gut microbiota *Weissella* also moved towards the third quadrant (Figure 2H). Cd significantly correlated with *Erysipelatoclostridium* (*r* = −0.06) and *Weissella* (*r* = 0.06); Pb significantly correlated with *Bacteroidaceae* (*r* = −0.06), *Bacteroides* (*r* = −0.06), *Intestinibacter* (*r* = −0.05), and *Butyricicoccus* (*r* = 0.03) (all *p* < 0.05) (Table S8, Figure 2I). Procrustes analysis also confirmed a strong correlation between the gut microbiota and heavy metals in dust (*r* = 0.22, *p* < 0.05) (Figure 2J).

Furthermore, we identified 11 co‐classification modules using Weighted Gene Co‐Expression Network Analysis (Table S9, Figure S12A). Environmental microbial modules affect differential gut microbiota (Figure S12B). Among them, the red, brown, and turquoise modules were significantly correlated with *Intestinibacter* and *Erysipelatoclostridium* (Figure S12C). The correlations of the modules are shown in Figure S12D. The hub‐microorganisms in the above three modules are presented in Figure S12E. *Intestinibacter* contributes to digestion, absorption, immune regulation, and the development of chronic diseases [17]. *Erysipelatoclostridium*, as a pathogen associated with zoonotic diseases, exhibits high infectivity and pathogenicity [18]. Notably, the direct correlation between heavy metals and the prementioned differential gut microbiota was weak. However, Fourth‐corner analysis identified a stronger correlation between them after environmental microorganisms joined, suggesting that these microorganisms act as the intermediary between heavy metals and gut microbiota. This connection can be explained partly by environmental microorganisms that can adsorb and accumulate heavy metals. Certain bacteria or fungi can bind to heavy metals through functional groups on their cell surfaces. This adsorption process increases the likelihood of heavy metals entering children's bodies directly. In the intestines, the cell structure of microorganisms may be disrupted, releasing the adsorbed heavy metals and subsequently interfering with the gut microbiota [19]. The environmental microbial community plays a critical role in disrupting gut microbiota by breaking down intestinal physical and chemical barriers, altering the microbiota structure on the intestinal epithelium [20]. The imbalance may impair gut microbial metabolic activity, increase heavy metal absorption in the gut, and amplify heavy metal toxicity. The interaction of environmental heavy metals and microorganisms jointly affects the stability and diversity of the gut microbiota of the exposed children.

This study has two limitations. First, only surface dust samples were analyzed, and airborne particulate matter was not considered. Second, lifestyle factors were not included as covariates, potentially influencing gut microbiota. In future, these limitations can be addressed by expanding the sampling locations, incorporating equipment to collect airborne particulate matter, and including children's lifestyle covariates in the analysis.

## CONCLUSION

This study indicates the presence of heavy metal pollution in schools, and its significant impact on the structure of the environmental microbial community. Pb and Cd were found to have pronounced effects on environmental microorganisms. Heavy metals and environmental microorganisms jointly affect the gut microbiota structure of exposed children, with environmental microorganisms playing a crucial intermediary role. Promoting healthy school development is vital to establish and enhance monitoring systems for heavy metal pollution, raise environmental health awareness, and protect children's well‐being.

## METHODS

This study utilized data from the Beijing Child Growth and Health Cohort, located in the urban area of a northeast district of Beijing, focusing on school environment measurements and children's fecal samples. We assessed the degree of environmental pollution by heavy metals concentration in the classroom dust as well the microbial community in the dust and categorized the fecal sample from the children according to the PLI: PLI ≤ 1 was defined as non‐polluted; 1 < PLI ≤ 2 as slight polluted; 2 < PLI ≤ 3 as moderate polluted; PLI > 3 as heavily polluted. Detailed procedures for sample collection, pollution assessment, sequencing protocol, and bioinformatic and statistical analysis approaches are available in the Supplementary Materials.

## AUTHOR CONTRIBUTIONS

**Yuchen Zou**: Writing—original draft; data curation; formal analysis; visualization. **Menglong Li**: Investigation; data curation; writing—review and editing. **Tuerxunayi Abudumijiti**: Data curation; writing—review and editing. **Huiming He**: Data curation. **Mengying Guan**: Data curation. **Yeerlin Asihaer**: Data curation. **Miao Li**: Data curation. **Nourhan M. Khattab**: Investigation; data curation. **Mushui Shu**: Investigation; methodology. **Yifei Hu**: Conceptualization; supervision; writing—review and editing.

## CONFLICT OF INTEREST STATEMENT

The authors declare no conflicts of interest.

## ETHICS STATEMENT

The ethics application (No. 2018SY82) was approved by the Research Ethics Committee of the Capital Medical University, and registered in Chinese Clinical Trial (ChiCTR2100044027).

## Supporting information


**Supplementary methodological explanation 1.** Sample collection and sequencing.
**Supplementary methodological explanation 2.** Heavy metal content detection.
**Supplementary methodological explanation 3.** Calculation method of heavy metal pollution and health assessment.
**Supplementary methodological explanation 4.** Statistical analysis, model introduction, and parameter selection for construction.
**Figure S1.** Results of heavy metal Geoaccumulation Index (GI) and Enrichment Factor (EF).
**Figure S2.** Quality of environmental sample sequencing and Alpha analysis results.
**Figure S3.** Analysis of environmental microbial abundance and trends.
**Figure S4.** Analysis of indoor microbial abundance and trends.
**Figure S5.** Microbial differences in environments with different sites.
**Figure S6.** Enrichment results of differential KEGG Orthology (KO) in KEGG map.
**Figure S7.** Quality of exposure children's fecal sample sequencing and Alpha analysis results.
**Figure S8.** Differences in gut microbiota among children exposed to different levels of pollution.
**Figure S9.** Results of eXtreme Gradient Boosting on heavy metals and environmental microorganisms.
**Figure S10.** The impact of heavy metals on microorganism functions.
**Figure S11.** The impact of heavy metals on gut microbiota and functions.
**Figure S12.** The correlation between environmental microorganisms and gut microbiota based on Weighted Gene Co‐Expression Network Analysis (WGCNA).

**Table S1.** Distribution of heavy metal content at each collection site (μg/g).
**Table S2.** Principal component analysis of heavy metals.
**Table S3.** Linear Discriminant Analysis (LDA) Effect Size analysis' significant results of environmental microorganisms in different sites.
**Table S4.** Fold change method's significant results of indoor microorganisms in different pollution levels.
**Table S5.** Enrichment results of differential Kyoto Encyclopedia of Genes and Genomes (KEGG) Orthology (KO) in KEGG map.
**Table S6.** Redundancy Analysis (RDA) results for heavy metals.
**Table S7.** Results of Mantel test.
**Table S8.** Fourth‐corner analysis results.
**Table S9.** The Weighted Gene Co‐Expression Network Analysis modules result.

## Data Availability

The data that supports the findings of this study are available in the supplementary material of this article. All the sequencing data have been deposited in the Genome Sequence Archive in the Beijing Institute of Genomics Data Center (https://ngdc.cncb.ac.cn/gsa/browse/CRA007292; https://ngdc.cncb.ac.cn/gsa/browse/CRA007303). The data and scripts used are saved in GitHub (https://github.com/HuLab908/iMeta2025-024). Heavy metal concentrations and microbial community profiles are provided within the main text and Supplementary materials. Supplementary materials (methods, figures, tables, graphical abstract, slides, videos, Chinese translated version, and update materials) may be found in the online DOI or iMeta Science http://www.imeta.science/.
