# Supplementary material for: The impact of school heavy metal exposure on children's gut microbiota: The mediating role of environmental microorganisms
Source: Imeta. 2025 Mar 25;4(2):e70021. doi: 10.1002/imt2.70021 (PMC11995171; doi:10.1002/imt2.70021)
Supplement: Supplementary file 1 — Supplementary methodological explanation 1. Sample collection and sequencing. Supplementary methodological explanation 2. Heavy metal content detection. Supplementary methodological explanation 3. Calculation method of heavy metal pollution and health assessment. Supplementary methodological explanation 4. Statistical analysis, model introduction, and parameter selection for construction. Figure S1. Results of heavy metal Geoaccumulation Index (GI) and Enrichment Factor (EF). Figure S2. Quality of environmental sample sequencing and Alpha analysis results. Figure S3. Analysis of environmental microbial abundance and trends. Figure S4. Analysis of indoor microbial abundance and trends. Figure S5. Microbial differences in environments with different sites. Figure S6. Enrichment results of differential KEGG Orthology (KO) in KEGG map. Figure S7. Quality of exposure children's fecal sample sequencing and Alpha analysis results. Figure S8. Differences in gut microbiota among children exposed to different levels of pollution. Figure S9. Results of eXtreme Gradient Boosting on heavy metals and environmental microorganisms. Figure S10. The impact of heavy metals on microorganism functions. Figure S11. The impact of heavy metals on gut microbiota and functions. Figure S12. The correlation between environmental microorganisms and gut microbiota based on Weighted Gene Co‐Expression Network Analysis (WGCNA). [file IMT2-4-e70021-s001.docx]

**Supporting information to “The impact of heavy metal exposure in campus environment on internal and external microorganisms”**

Yuchen Zou^1^, Menglong Li^1^, Tuerxunayi Abudumijiti^1^, Huiming He^1^, Mengying Guan^1^, Yeerlin Asihaer^1^, Miao Li^1^, Nourhan M. Khattab^1^, Mushui Shu^2^, Yifei Hu^1*^

^1^Department of Child, Adolescent Health and Maternal Care, School of Public Health, Capital Medical University, Beijing, 100069, China.

^2^Institute of Urban Safety and Environmental Science, Beijing Academy of Science and Technology, Beijing 100054, China.

Correspondence：huyifei@yahoo.com (Yifei Hu)

Contents

Supplementary methodological explanation 1. Sample collection and sequencing. 1

Supplementary methodological explanation 2. Heavy metal content detection. 2

Supplementary methodological explanation 3. Calculation method of heavy metal pollution and health assessment. 3

Supplementary methodological explanation 4. Statistical analysis, model introduction, and parameter selection for construction. 6

Figure S1. Results of heavy metal Geoaccumulation Index (GI) and Enrichment Factor (EF). 10

Figure S2. Quality of environmental sample sequencing and Alpha analysis results. 11

Figure S3. Analysis of environmental microbial abundance and trends. 12

Figure S4. Analysis of indoor microbial abundance and trends. 13

Figure S5. Microbial differences in environments with different sites. 14

Figure S6. Enrichment results of differential KEGG Orthology (KO) in KEGG map. 15

Figure S7. Quality of exposure children's fecal sample sequencing and Alpha analysis results. 16

Figure S8. Differences in gut microbiota among children exposed to different levels of pollution.

17

Figure S9. Results of eXtreme Gradient Boosting on heavy metals and environmental microorganisms. 18

Figure S10. The impact of heavy metals on microorganism functions. 19

Figure S11. The impact of heavy metals on gut microbiota and functions. 20

Figure S12. The correlation between environmental microorganisms and gut microbiota based on Weighted Gene Co-Expression Network Analysis (WGCNA). 21

Supplementary methodological explanation 1. Sample collection and sequencing.

1. Sample collection

Fecal samples from children were collected in 2019 (detailed in the previous study [1]) using fecal occult blood test card (BA-2020B, Wuhan Zhuhai Beisuo Biotechnology Co., Ltd.). All samples were stored in -80℃ refrigerator. A total of 126 samples were performed 16S rRNA and obtained gut microbiota information for children exposed slight (*n* = 4), moderate (*n* = 39), and heavy pollution (*n* = 83). To further explore the functional information, 18 children exposed moderate (5) and heavy pollution (13) were selected for meta gene sequencing.

Dust samples were collected in February 2019 from 67 classrooms, each classroom having three sites for sample collection, including indoor, outdoor, and air-conditioning. The classrooms include standard teaching spaces for subjects like mathematics and literature, as well as specialized art classrooms for activities such as music and dance. Using an individual brush, dust was swept on tin foil at each site. Indoor dust samples included all dust on the horizontal surface above the lampshade, blackboard, the door frame, and the window inner frame; outdoor dust included all dust of the inside window slot and the outside window slot and sill surface. Air-conditioning dust was the accumulated dust on the air-conditioning filter. Each tin foil was carefully stacked, labeled with a number. Indoor samples were classified into three pollution levels per Pollution load index (PLI): slight, moderate, and heavy. A total of 41 indoor, 6 outdoor, and 17 air-conditioning samples from ordinary classrooms were extracted for metagenomic sequencing to obtain environmental microbial information.

2. Dust samples sequencing

Metagenomic sequencing performed on dust samples. Randomly break the qualified samples into 350bp fragments, prepared the library and qualified libraries (effective concentration > 3nM) were sequenced using Illumina PE150. Performed quality control and host filtering on the raw data, then obtained clean data. After assembling Metagenome, MetaGeneMark was used for gene prediction based on scaftigs of a single sample, then redundancy removal to construct a gene catalogue. Compared the gene catalogue with the MicroNR library to obtain each gene species annotation information; combined it with the gene abundance table to obtain species abundance tables for different classification levels. Performed functional annotation and abundance analysis of Kyoto Encyclopedia of Genes and Genomes (KEGG), homologous gene clusters (eggNOG), and carbohydrate enzymes (CAZy).

3. Fecal samples sequencing

Fecal samples performed 16S rRNA sequencing and metagenomic sequencing. Performed PCR amplification, library preparation, library quality inspection on the fecal samples. The Illumina Hiseq 2500 platform was used for sequencing. Double-ended PE reads were spliced into target region sequences, followed by quality control filtering. Filtered sequences were compared with the reference database, and the chimeric sequences were removed to generate the final optimized sequences. We performed Operational Taxonomic Unit clustering analysis and taxonomy annotation on optimized sequences, and the Metagenomic sequencing was performed on the Illumina HiSeq platform. The processing flow of the raw data was the same as dust sample sequencing.

Supplementary methodological explanation 2. Heavy metal content detection

The concentration (μg/g) of cadmium (Cd), cobalt (Co), chromium (Cr), copper (Cu), manganese (Mn), nickel (Ni), lead (Pb), strontium (Sr), and vanadium (V) were determined using inductively coupled plasma mass spectrometry (ICP-MS, ICPE-9000, Shimadzu, Japan). After drying for 24hrs, samples filtered through a 200-mesh sieve to remove impurities. Add each sample, 0.010 ml internal standard (1000 µg/ml yttrium standard solution) and 10ml of digestion solution (nitric acid: perchloric acid: hydrofluoric acid = 8:1:1) into a PTFE digestion tube, covered with a reflux funnel. Placed the tube into the digestion apparatus, maintained at 170℃ for 3hrs, removed the reflux funnel, then maintained until almost dry. After cooling to room temperature, dilute to 10ml with 10% nitric acid for testing. The above metals were prepared into standard series of 0.05, 0.1, 0.5, 1, 5, and 10μg/ml using 10% nitric acid. Measured the standard series to ensure that the linear r-value of the standard curve was greater than 0.99. Inserted one quality control sample every ten samples, with an absolute relative error of less than 10%. The limit of detection (LOD, μg/L) and limit of quantification (LOQ, μg/L) were also calculated by ICP-MS (Table S1).

Table S1. LOD, LOQ, and detection rate (DR) of heavy metals.

| Metals | LOD (μg/L) | LOQ (μg/L) | DR (%) |
| --- | --- | --- | --- |
| Cd | 0.94 | 3.10 | 100 |
| Co | 0.24 | 0.79 | 100 |
| Cr | 0.57 | 1.90 | 100 |
| Cu | 3.58 | 12.0 | 100 |
| Mn | 0.13 | 0.42 | 100 |
| Ni | 1.40 | 4.70 | 100 |
| Pb | 1.93 | 6.40 | 100 |
| Sr | 0.47 | 1.60 | 100 |
| V | 0.10 | 0.34 | 100 |

Supplementary methodological explanation 3. Calculation method of heavy metal pollution and health assessment

This study used the Geoaccumulation Index (GI), Enrichment Factor (EF) to evaluate heavy metal pollution; used PLI to identify the comprehensive pollution level. The U.S. Environmental Protection Agency human exposure risk assessment method was also used for health risk assessment of exposed children [2]. The calculation formula, parameter selection, and level classification were described as follows.

1. GI

The GI is widely applied for the risk assessment of heavy metal pollution caused by human activities in different environmental media. The formula is as follows:

$I_{geo}=\log_{2} (C_{n}/1.5B_{n})$ (1)

*C_n_* is the measured heavy metal content (μg/kg), *B_n_* is the heavy metal background value (μg/kg), which is the background value in Beijing, and 1.5 is the correction index. The evaluation criteria are:

Table S1. Evaluation criteria for GI

| Level | GI value | Pollution levels |
| --- | --- | --- |
| Ⅰ | GI ≤ 0 | Non pollution |
| Ⅱ | 0 < GI ≤ 1 | Slight pollution |
| Ⅲ | 1 < GI ≤ 2 | Slight-moderate pollution |
| Ⅳ | 2 < GI ≤ 3 | Moderate pollution |
| Ⅴ | 3 < GI ≤ 4 | Moderate-heavy pollution |
| Ⅵ | 4 < GI ≤ 5 | Heavy pollution |
| Ⅶ | GI > 5 | Serious pollution |

In this study, the metals background values in Beijing were selected as follows: cadmium (Cd) 0.09, cobalt (Co) 10, chromium (Cr) 58, copper (Cu) 20, manganese (Mn) 521, nickel (Ni) 25, lead (Pb) 19, and vanadium (V) 71 [3,4].

2. EF

EF is an important parameter for evaluating the human activities impaction on the enrichment of heavy metals. Normalize the test sample metals by the reference metal as a reference standard. The reference metal is required to be less susceptible to the influence of the environment and analytical testing process, and its properties are relatively stable. In this study, strontium (Sr) was used as the reference metal [5]. The formula is as follows:

$EF=\left[ C_{x}/C_{ref} \right]_{s}/\left[ C_{x}/C_{ref} \right]_{B}$ (2)

*C_x_* is the measured heavy metal content (μg/g), *C_ref_* is the reference metal content (μg/g), *S* and *B* represent the sample and background values (μg/g), respectively. The evaluation criteria are:

Table S2. The evaluation criteria for EF

| Level | EF value | Pollution levels |
| --- | --- | --- |
| Ⅰ | EF < 2 | Non-slight pollution |
| Ⅱ | 2 ≤ EF < 5 | Moderate pollution |
| Ⅲ | 5 ≤ EF < 20 | Moderate-heavy pollution |
| Ⅳ | 20 ≤ EF < 40 | Heavy pollution |
| Ⅴ | EF ≥ 40 | Serious pollution |

3. PLI

PLI is the comprehensive evaluation method in heavy metal pollution research, and it can avoid the influence of pollution index addition relationship on the evaluation results, can better evaluate the anthropogenic heavy metals pollution. The formula is as follows:

$PLI=\sqrt[n]{\prod_{i=1}^{n} C_{i}/B_{i}}$ (3)

*C_i_* represents the measured heavy metal content (μg/g), and *B_i_* represents the corresponding background value (μg/g). The evaluation criteria are:

Table 3. The evaluation criteria for PLI

| Level | PLI value | | Pollution levels | |
| --- | --- | --- | --- | --- |
| Ⅰ | | PLI ≤ 1 | | Non pollution |
| Ⅱ | | 1 < PLI ≤ 2 | | Slight pollution |
| Ⅲ | | 2 < PLI ≤ 3 | | Moderate pollution |
| Ⅳ | | PLI > 3 | | Heavy pollution |

4. Heavy metal health risk assessment

The health risk assessment of heavy metals on exposed students adopts the U.S. Environmental Protection Agency (EPA) human exposure risk assessment method [2]. The exposure pathways of heavy metal that threat to the health of students can be divided into three types: hand-to-mouth ingestion, inhalation, and dermal contact. The calculation formulas for the average daily exposure dose for different exposure pathways are as follows:

Hand-to-mouth ingestion：

${ADD}_{ing}=C\times\frac{IngR\times EF\times ED}{BW\times AT}\times{10}^{-6}$ (4)

Inhalation：

$ADD_{inh}=C\times\frac{InhR\times EF\times ED}{PEF\times BW\times AT}$ (5)

Dermal contact：

${ADD}_{dermal}=C\times\frac{SA\times SL\times ABSd\times EF\times ED}{BW\times AT}\times{10}^{-6}$ (6)

*C* is the heavy metal measured concentration (mg/kg). *ADD_ing_, ADD_inh_*, and *ADD_dermal_* represent the average daily doses in different routes (mg/kg/day). *IngR* represents the hand-to-mouth ingestion frequency: 200mg/kg/day; *InhR* represents the inhalation frequency: 100mg/day. *EF* is the exposure frequency, and the exposure frequency of students is estimated about 190 days/year; *ED* is the exposure duration, and Beijing primary school is a six-year system, so the exposure period is 6 years. *SA* is the exposed skin area (m^2^), calculated using the Xu Wensheng formula: *SA*_male_ = 0.0057 × height (cm) + 0.0121 × weight (kg) － 0.0882; *SA*_female_ = 0.0073 × height (cm) + 0.0127 × weight (kg) － 0.2106. *SL* is skin adhesion (skin conductance level): 0.2 mg/cm^2^/hr. *ABSd* is the dermal absorption fraction, and each metal skin absorption factor are 0.001. *PEF* is the particulate matter emission factor, and the EPA has published a value of 1.36 × 109 m^3^/kg. *BW* is the body weight of children exposed to heavy metals collected in the same year. *AT* is the exposure average time, for non-carcinogenic risks *AT* = *ED* × 365 days.

The formula for calculating the non-carcinogenic hazard index of heavy metal is as follows:

$HQ=\frac{{ADD}_{ing/inh/dermal}}{{RfD}_{ing/inh/dermal}}$ (7)

$HI={HQ}_{ing}+{HQ}_{inh}+{HQ}_{dermal}$ (8)

*ADD* refers to the average daily doses (mg/kg/day); *RfD* (reference dose) is the safe reference dose (mg/kg/day); *HQ* is the hazard quotient for individual pollutants in each exposure pathway non-carcinogenic substances. *HI* is the total non-carcinogenic hazard index. When *HI* ≤ 1, it is considered that the risk is small or can be ignored; on the contrary, it is considered that there is a non-carcinogenic risk.

Supplementary methodological explanation 4. Statistical analysis, model introduction, and parameter selection for construction

The heavy metal concentration is expressed as mean ± standard deviation (SD). One-way Analysis of Variance (ANOVA) and Kruskal-Wallis test were used to compare the differences between groups. The Bonferroni correction was used for post-hoc analysis in ANOVA and False Discovery Rate (FDR) was applied for correction after the Kruskal-Wallis test. Alpha and Beta analyses were used to analyze microbial diversity. Differential microorganisms were identified by Linear Discriminant Analysis Effect Size (LEfSe) and Fold Change (FC) method.

The impact of heavy metals on species or functional composition was analyzed by Redundancy Analysis (RDA) and Shapley Additive Explanations (SHAP) [based on eXtreme Gradient Boosting (XGboost)]. The correlation between heavy metals and environmental microorganisms or gut microbiota was demonstrated by Spearman correlation analysis and Mantel’s test; the association between environmental microorganisms and gut microbiota was explored by Weighted Gene Co-Expression Network Analysis (WGCNA); the relationship between all three was identified using Relative Length of the Quadrant (RLQ) and Fourth-corner analysis. Statistical significance was determined using a two-sided *p* < 0.05, and the bioinformatics analysis *p* was subjected to 999 permutation tests. The bioinformatics analysis in this study was using R software (4.4.1ver).

1. Alpha analysis

Alpha analysis was evaluated using Shannon and Simpson indexes. The Shannon and Simpson indexes covers the species richness and evenness of the community, the larger value, the higher the community diversity. The calculation formula is:

$H_{shannon}=-\sum_{i=1}^{S_{obs}} \frac{n_{i}}{N}\ln\frac{n_{i}}{N}$ (1)

$D_{Simpson}=1-\frac{\sum_{i=1}^{S_{obs}} n_{i}(n_{i}-1)}{N(N-1)}$ (2)

*S_obs_* represents the actual number of Operational Taxonomic Units (OTUs) measured, *n_i_* is the OTUs containing number of *i* sequences, and *N* is the total number of sequences. We used the Vegan package for computation.

2. Bate analysis

Beta analysis was calculated by Vegan package.

2.1 Principal Component Analysis (PCA)

PCA is a statistical method that can transform a set of potentially correlated variables into a set of linearly uncorrelated variables through orthogonal transformation, known as principal components. The steps of PCA usually include: standardized data, calculate covariance matrix, calculate eigenvalues and eigenvectors, select principal components, transform data.

2.2 Principal Co-ordinates Analysis (PCoA)

PCoA is an unconstrained data dimensionality reduction analysis method that can be used to study the similarity or dissimilarity of sample community composition. PCoA allows the use of non-Euclidean distances, and in this study, Bray-Curtis distance was used to describe the similarity between communities.

2.3 Permutation Multivariate Analysis of Variation (PERMANOVA)

PERMANOVA is a multivariate analysis of variance method used to compare discrete data between multiple groups. It is to conduct permutation tests based on distance matrices (we caculated Bray-Curtis distance), calculate *p* through chi-square distribution approximation, and perform 999 permutation tests to evaluate the significance of inter group differences.

2.4 Permutational Multivariate analysis of Dispersion (PERMDISP)

The purpose of PERMDISP is to examine whether there are significant differences in dispersion among different groups, which can reflect the variability of individuals within the group. It is usually used in conjunction with PERMANOVA. The model calculates the dispersion (we caculated Bray-Curtis distance) of each sample relative to its group centroid or spatial median, and uses 999 permutation tests to evaluate whether the differences in dispersion between groups are statistically significant. If there is no statistical difference, it proves that the differences between different groups are not due to individual dispersion, but rather differences in the centroid distance of the groups.

3. Differential analysis

3.1 LEfSe

LEfSe is a statistical method used in bioinformatics. Firstly, the non-parametric Kruskal-Wallis rank sum test is used to identify features with significant differences in abundance between different groups. It uses Wilcoxon rank sum test to evaluate the consistency of significantly different features between different groups. Finally, Linear Discriminant Analysis (LDA) was used to estimate the magnitude of the impact of each feature abundance on the inter group difference effect. The higher the LDA score, the greater the contribution of the feature in distinguishing different groups. LDA score > 2 and *p* < 0.05 will be identified as differential microorganism. This study used the trans-diff $ new () function in the Microeco package for calculations, with all parameters set to default values. Parameters details please refer to: <https://www.rdocumentation.org/packages/microeco/versions/0.7.1/topics/trans_diff>.

3.2 FC methods

The calculation method for the FC is as follows:

$FC=\frac{Mean express level in group1}{Mean express level in group2}$ (3)

This study used the Limma package to calculate the FC in microbial abundance, and applied Bayesian statistical methods through the eBayes () function to calculate the statistical significance of differential expression. Microorganism with |Log_2_FC| > 2.5 and *p* < 0.05 will be identified as differential microorganism.

4. Analysis of heavy metal effects

4.1 RDA

It is a multivariate statistical method that combines regression analysis and PCA to explore the relationship between microorganism abundance and environmental factors. Determine whether RDA is suitable by Detrended Correspondence Analysis. If the size of the Length of Grade Axis1 > 4.0, Canonical Correlation Analysis (CCA) should be selected; if it is between 3.0 to 4.0, both RDA and CCA can be selected; if it < 3.0, RCA should be selected. This study requires RDA. Calculating the inflation factors (IFs) value of heavy metals, if the heavy metal’s IFs > 10, the metals cannot be included. This study used the Vegan package to performed RDA and applied 999 permutation tests to evaluate the statistical significance of heavy metals.

4.2 XGboost and SHAP

XGboost is an optimization algorithm based on the Gradient Boosting framework, which not only focuses on reducing training errors, but also introduces regularization terms to control model complexity and prevent overfitting. The objective function is:

$L(\theta)=\sum_{i=1}^{n} l(y_{i},\hat{y}_{i}^{\left( t \right)})+\Omega(f)$ (4)

*l* is the loss function, Ω is a regularization term used to penalize model complexity. XGboost adopts column block minimization technique, which only traverses a part of the features in each iteration process, greatly reducing the computational complexity. We used xgboost packages, set the training and testing sets in a 4:1 ratio. Set the parameter selection range to nrounds = c (75, 100, 150), colsample_bytree = 1, min_child_weight = 1, eta = c (0.01, 0.1, 0.3), gamma = c (0.5, 0.25, 0.1), subsample = 0.5, max_depth = c (2, 3, 4), choose the best parameter by five Cross-Validation tests. SHAP is a machine learning model interpretation method. It is based on the Shapley value in game theory, assigning importance values to each feature of the model to explain the prediction process of the model. Based on the XGboost results, we used shapviz packages to perform it.

4.3 Mantel test

The basic principle of Mantel test is to calculate the correlation coefficient (we used Pearson's product-moment correlation) between two matrices. The null hypothesis of the test is that there is no correlation between two matrices. Through 999 permutation tests, the rows and columns of one of the matrices are randomly permuted to maintain the symmetry of the matrix and keep the zero-diagonal unchanged, and the correlation coefficients between the permuted matrices are recalculated to generate a distribution of correlation coefficients. Then, compare the original observed correlation coefficients with the correlation coefficient distribution generated by permutation to determine whether their correlation has statistical significance.

5. RLQ and Fourth-corner analysis

RLQ analysis is an ecological research method used to explore the relationship between traits and environmental variables. This method analyzes how species traits respond to environmental changes by combining the environmental factor matrix (R), abundance matrix (L), and trait matrix (Q) of the species. In this study, Q was the gut microbiota abundance matrix. The steps of RLQ analysis usually include: step1, performing Correspondence Analysis (COA) on the L matrix. Step2, perform PCA on the Q matrix using the exercise weights obtained in step1. Setp3, perform PCA on the R matrix using the column weights obtained in step1. Perform RLQ analysis, compare the covariance of three independent analyses, and reveal the main relationships between traits, species abundance, and environmental factors through Co Inertia Analysis.

The core idea of the Fourth-corner analysis is to combine these three matrices and correlate L matrix with Q matrix and R matrix through weighting. Based Polynomial to study the relationship (Pearson's product-moment correlation) between species characteristics and environmental variables through a weighted matrix. Use False Discovery Rate to evaluate whether these relationships have statistical significance.

6. WGCNA

We used WGCNA to calculate the correlation between each gene module and the gut microbiota to evaluate the correlation between the module and the gut microbiota. WGCNA is a method used to analyze the interrelationships between genes in gene expression data. It uses Pearson correlation to calculate the correlation between each pair of genes, and calculates the weight of each edge in the network using a weighted method based on correlation. WGCNA identify gene modules through hierarchical clustering analysis, which involves clustering closely connected genes in the network together to form modules. In this study used WGCNA package for analysis. Due to the large number of genes, we selected the top 25% of genes with variance. Through sample clustering inspection, we removed one outlier sample. The power was determined to be 10 using the pickSoftThreshold () function. The maximum number of module genes was set to 6000 and the minimum was 100.

References

1. Liang, Tian, Dan Li, Jiawulan Zunong, Menglong Li, Nubiya Amaerjiang, Huidi Xiao, Nourhan M Khattab, Sten H Vermund, Yifei Hu. 2022. “Interplay of lymphocytes with the intestinal microbiota in children with nonalcoholic fatty liver disease.” *Nutrients* 14: 4641. <https://doi.org/10.3390/nu14214641>

2. Hou, Shengnan, Na Zheng, Lin Tang, Xiaofeng Ji, Yunyang Li, Xiuyi Hua. 2019. “Pollution characteristics, sources, and health risk assessment of human exposure to Cu, Zn, Cd and Pb pollution in urban street dust across China between 2009 and 2018.” *Environment International* 128: 430-437. <https://doi.org/10.1016/j.envint.2019.04.046>

3. Cheng, Hangxin, Kuo Li, Min Li, Ke Yang, Fei Liu, Xiaomeng Chen. 2014. “Background and baseline values of soil chemical elements in Chinese cities.” *Earth Science Frontiers* 21: 42. [in Chinese]

4. Chen, Tongfu, Yuanming Zheng, Huang Chen, Guodi Zheng. 2004. “Systematic Study on Background Values of Soil Heavy Metal Content in Beijing.” *Environmental Sciences* 25: 6. [in Chinese]

5. Loska, Kzysztof, Danuta Wiechuła, Irena Korus. 2004. “Metal contamination of farming soils affected by industry.” *Environment International* 30: 159-165. <https://doi.org/10.1016/s0160-4120(03)00157-0>

Figure S1. Results of heavy metal Geoaccumulation Index (GI) and Enrichment Factor (EF).

(A) The GI results of heavy metals. (B) Distribution of GI at different sites. (C) Statistical differences between different sites. (D) The EF results of heavy metals. (E) Distribution of EF at different sites. (F) Statistical differences between different sites. The *p* of Kruskal-Wallis test were corrected by FDR. ^*^*p* < 0.05, ^**^*p* < 0.01, ^***^*p* < 0.001. Air-conditioning was the air-conditioner filter sample.

Figure S2. Quality of environmental sample sequencing and Alpha analysis results.

(A) Species accumulation curves. (B) Rank abundance curves. (C) Alpha diversity analysis of environmental microorganisms. (D) Alpha diversity analysis of indoor microorganisms. Air-conditioning was the air-conditioner filter sample.

Figure S3. Analysis of environmental microbial abundance and trends.

On the figure left is a heatmap of each sample environmental microorganism’s abundance after standard. Samples from different sites and different clustering modules are represented by different colors, and typical trending microorganisms in the modules are labeled with corresponding colors on the left side of the heatmap. On the figure right is a heatmap of the average environmental microbial abundance of three sites, with different clustering modules represented by different colors. The module expression trend line is labeled on the right side of the heatmap, and the trend typical microorganisms are labeled on the left side. Air-conditioning was the air-conditioner filter sample.

Figure S4. Analysis of indoor microbial abundance and trends.

On the figure left is a heatmap of each sample indoor microorganism’s abundance after standard. Samples from different pollution levels and different clustering modules are represented by different colors, and typical trending microorganisms in the modules are labeled with corresponding colors on the left side of the heatmap. On the figure right is a heatmap of the average indoor microbial abundance of three pollution levels, with different clustering modules represented by different colors. The module expression trend line is labeled on the right side of the heatmap, and the trend typical microorganisms are labeled on the left side.

Figure S5. Microbial differences in environments with different sites.

(A) The environmental microorganism’s composition in different sites at the phylum level (left), and the species level (right). (B) Principal Co-ordinates Analysis (PCoA) at the phylum level (upper) and species level (lower) of environmental microorganisms, with a 95% confidence interval (CI) for shadows. (C) The Linear Discriminant Analysis Effect Size (LEfSe) analysis results for microorganism. The LEfSe analysis results for Kyoto Encyclopedia of Genes and Genomes (KEGG) (D), homologous gene clusters (eggNOG) (E), and carbohydrate enzymes (CAZy) (F). The yellow line represents the number of annotated genes displayed on the upper X-axis. ^*^*p* < 0.05, ^**^*p* < 0.01, ^***^*p* < 0.001. Air-conditioning was the air-conditioner filter sample.

Figure S6. Enrichment results of differential KEGG Orthology (KO) in KEGG map.

Display five KEGG maps showing significant differences among three pollution levels. The color represents the Reporter value for each KO enrichment. Yellow indicates significant up-regulated enrichment, green indicates significant down-regulated enrichment, gray indicates no significance, and white indicates no enrichment. The red border highlights the differential gut microbiota KO that this study focuses on, while the blue border highlights the differential environmental microbiota KO. The *p* was corrected by FDR.

Figure S7. Quality of exposure children's fecal sample sequencing and Alpha analysis results.

(A) Species accumulation curves. (B) Rank abundance curves. (C) Shannon index of gut microbiota. (D) Simpson index of gut microbiota.

Figure S8. Differences in gut microbiota among children exposed to different levels of pollution.

(A) Heavy metals pose non carcinogenic hazard index. (B) The gut bacterial community composition in two groups at the phylum level (left), and the genus level (right). (C) PCoA at the phylum level (upper) and genus level (lower) of gut bacteria. (D) Slight pollution vs moderate pollution differential gut microbiota (upper) and slight pollution vs heavy pollution differential gut microbiota (lower). Orange up-triangles means the significant up-regulated gut microbiota. (E) The LEfSe analysis results for each group. (F) Functional enrichment results. ^*^*p* < 0.05, ^**^*p* < 0.01, ^***^*p* < 0.001.

Figure S9. Results of eXtreme Gradient Boosting on heavy metals and environmental microorganisms.

Phylum-level (left) (A) and species-level (right) (B) of environmental microorganisms predict interpretability and Shapley Additive Explanations (SHAP) interactions. Phylum-level (left) (C) and species-level (right) (D) of indoor microorganisms predict interpretability and SHAP interactions. In predict interpretability plot, the length of the arrow measures the contribution of each feature to the model prediction; positive values (pink) indicate that the feature has increased the predicted value, while negative values (blue) indicate decreased. Above the arrow are the heavy metals characteristic values. F(x) is the sum of all feature contributions, and E[f(x)] is the baseline prediction value of the model without any feature information.

Figure S10. The impact of heavy metals on microorganism functions.

(A) RDA results of the KEGG (upper) and CAZy (lower) of environmental microorganisms. (B) RDA results of the KEGG (upper) and CAZy (lower) of indoor microorganisms. The title is the DCA result of the longest axis of the trend; the ellipse is the 95% CI; variables with high correlation tend to have consistent arrow directions, and the length of the arrow represents the contribution of the ranking, red arrows are significant heavy metals; the *p* is replaced with 999 permutation tests. (C) Mantel test of heavy metals and environmental microorganisms KEGG level1 items. (D) Mantel test of heavy metals and indoor microorganisms KEGG level1 items. ^*^*p* < 0.05, ^**^*p* < 0.01, ^***^*p* < 0.001. The lower triangle represents the Pearson autocorrelation of heavy metals; the line width is the Mantel test *r* and the line color is Mantel test *p*. Air-conditioning was the air-conditioner filter sample.

Figure S11. The impact of heavy metals on gut microbiota and functions.

(A) RDA results of the phylum-level (upper) and genus-level (lower) of gut microbiota. (B) SHapley Additive exPlanations analysis of heavy metals and gut microbiota Shannon index. The number on the right side of the Y-axis represents the importance index; red indicating high heavy metal exposure dose and blue indicating low. (C) Spearman correlation between differential gut microbiota and heavy metals. ^*^*p* < 0.05, ^**^*p* < 0.01, ^***^*p* < 0.001. (D) RDA results of the KEGG (upper) and CAZy (lower) of gut microbiome. (E) Mantel test of heavy metals and gut microbiome KEGG level1 items.

Figure S12. The correlation between environmental microorganisms and gut microbiota based on Weighted Gene Co-Expression Network Analysis (WGCNA).

(A) WGCNA cluster tree diagram of environmental microorganisms, each branch represents an environmental microorganism and the color represents the module of genes assigned. (B) Cluster tree diagram of differential gut microbiota, each branch representing a sample and colors indicating the gut microbiota abundance. (C) The correlation results between modules and differential gut microbiota, corresponding modules' correlation coefficients and *p* are shown in the blocks. (D) Autocorrelation between modules. (E) The network of hub microorganisms in key modules (Red, Brown, Turquoise), where each point represents a hub-microorganism and the color represents the module.
